# The role of balanced time perspective mediating the relationship between mindfulness as a trait and life satisfaction in Catalan University students

**DOI:** 10.1007/s12144-022-03795-4

**Published:** 2022-11-09

**Authors:** Queralt Ballabrera, Marc Pérez-Burriel

**Affiliations:** 1 University of Girona, Girona, Spain; 2 Department of Psychology, University of Girona, Girona, Spain

**Keywords:** Time Perspective, Balanced time perspective, Mindfulness, Life satisfaction

## Abstract

The objective of the present study was to replicate previous studies by examining the potential connections between mindfulness as a trait, life satisfaction and time perspectives (TP) and to analyze if balanced time perspective (BTP) could act as a possible mediator between mindfulness and life satisfaction in students from the University of Girona (Catalonia, Spain). Data was collected from 341 Catalan university students aged 18–52 (77.1% women; mean age = 23.62) who completed Zimbardo Time Perspective Inventory (ZTPI), the Mindfulness Awareness Scale (MAAS) and the Satisfaction with Life Scale (SWLS) to assess their Temporal Perspective, Mindfulness as a trait and Life Satisfaction respectively. Balanced time perspective (BTP) was calculated using the deviation index of BTP (DBTP). Results showed significant relationships between the various temporal dimensions, life satisfaction, and mindfulness as a trait, being the most significant associations with balanced temporal perspective. On the other hand, results also suggested that BTP may act as a mediator between mindfulness as a psychological trait and life satisfaction, confirming previous studies conducted in different countries. In conclusion, findings could highlight the importance of regulating one’s attention in order to balance time perspectives, which in turn would translate into improved life satisfaction.

**Supplementary Information:**

The online version contains supplementary material available at 10.1007/s12144-022-03795-4.

The study of time has caught the interest of various disciplines throughout history. Philosophy, biology, physics, and psychology have long debated about this phenomenon, and the desire to understand the mechanisms by which people experience and perceive time has been pursued since antiquity (Zimbardo & Boyd, 2008).

Within the field of psychology, one aspect that has become relevant among researchers in recent years is the notion of *Time Perspective* (TP). Much of the research on TP can be attributed to psychologists Philip Zimbardo and John Boyd, who developed the first multidimensional and psychometrically robust measurements of TP (Stolarski et al., 2015; Zimbardo et al., 2012; Zimbardo & Boyd, 1999). These authors, based on the work of Kurt Lewin, conceptualize TP as “the often-nonconscious process whereby the continual flows of personal and social experiences are assigned to temporal categories, or time frames, that help to give order, coherence, and meaning to those events” (Zimbardo & Boyd, 1999, p. 1271). Although we can think about our past, present, and future, due to the limitations of our attentional capacity, we can only focus on one TP at a given time, leaving the other two out of our focus of attention. In other words, if one concentrates on one’s future, one will likely ignore one’s past, leaving few cognitive resources available to control one’s present situation. Similarly, concentration on the present reduces the resources available to consider the future consequences of present behavior, and so on (Stolarski et al., 2015).

TP reflects our attitudes, beliefs, and values in relation to time and is believed to be heavily influenced by socialization, education, culture, and other situational factors (Boniwell et al., 2010). It has been linked to various aspects of human functioning, including health-related behaviors (Henson et al., 2006), risk-taking (Boyd & Zimbardo, 2005), academic performance (Mello & Worrell, 2006), emotional intelligence (Stolarski et al., 2011), and age (Laureiro-Martinez et al., 2017; Zimbardo & Boyd, 1999) defined five time frames as individual temporal profiles (see Table 1).

**Table 1:** *Time frames defined by* Zimbardo and Boyd (1999*)*

Time frames	Code	Description
**Past Negative**	**PN**	Related to a generally negative and aversive view of the past, which may be the result of the actual experience of unpleasant or traumatic events, a negative reconstruction of benign events, or a mixture of both.
**Past Positive**	**PP**	Reflecting a warm and sentimental attitude towards the past.
**Present hedonistic**	**PH**	Related to a focused attitude toward obtaining pleasure, impulsivity, and little concern for future consequences.
**Present Fatalistic**	**PF**	Related to the belief that the future is predestined, cannot be influenced through individual action, and cannot be changed in any way in the present moment.
**Future**	**F**	Related to behavior dominated by the struggle to achieve future goals and rewards.

A revised conceptualization included a new dimension, the *Future Negative*, characterized by a behavior dominated by future anxiety as well as a focus on potential threats that may occur (Carelli et al., 2011).

In addition to the abovementioned dimensions, Zimbardo and Boyd defined the concept of *Balanced Time Perspective* (BTP), which refers to an individual’s ability to flexibly shift between various time frames depending on task characteristics, demands, situational considerations, and personal resources, thus allowing for effective adaptation to changing environmental demands (Stolarski, Zajenkowski et al., 2020, Stolarski, Wojciechowski & Matthews, 2020; Wiberg et al., 2017; Zimbardo & Boyd, 1999).

Over the years, several researchers have empirically operationalized the concept of BTP in various ways (correlated studies, cluster analysis, or a combination of quantitative and qualitative methodologies, among others) (Wiberg et al., 2017). One of these proposals—and the one considered in this study—was carried out by Stolarski, Bitner, and Zimbardo (Stolarski et al., 2011), who introduced an indicator—the deviation from the balanced time perspective (DBTP) coefficient—based on the Euclidean distance between an individual’s time perceptions and the optimal time perspective profile as stated by Zimbardo and Boyd (2008). This method is one of the most widely used and is supported by empirical evidence (Zhang et al., 2013).

## Time perspective, satisfaction with life and wellbeing

In recent years, many studies interested in the connection between TP and subjective well-being components have grown. One of these components is *Life Satisfaction*, conceptualized as “a cognitive, global appraisal that people make when considering their contentment with their life as a whole or in regard to specific domains of life such as family, environment, friends, and self” (Suldo & Huebner, 2006, p. 180).

Several studies have found associations between different time perspectives and life satisfaction. Specifically, Past Negative has demonstrated the most significant negative association (Boniwell et al., 2010; Drake et al., 2008; Pallini et al., 2018; Seema & Sircova, 2013; Stolarski et al., 2016). On the other hand, several studies have shown that BTP has stronger relationships with well-being measures than any other time perspective measurement (Zhang et al., 2013). Thus, BTP would be related to the highest levels of well-being and life satisfaction (Stolarski & Matthews, 2016; Zhang et al., 2013; Boniwell et al., 2010; Drake et al., 2008).

On the other hand, Durayappah (2011) proposed a temporal model of subjective well-being, called the 3P Model. This model is presented as a parsimonious, unifying theory of subjective well-being, that concludes that “a happy event in one’s life is meaningful when it is meaningful not just to our current self but also has meaning for our past self and future self” (p. 710). This is to say, shortening the link between the subjective well-being and the satisfaction with life through the integration of this event in the life [meaningful] course.

## The role of mindfulness in time perspective

At the turn of the century there was an outbreak in the interest in meditation practices and their adaptations in Western contexts, such as *Mindfulness*, that continues to grow year after year. Although the reasons behind this phenomenon are unclear, Mindfulness is positioning as an increasingly accepted practice in everyday life as well as in clinical practice and psychosocial interventions (Crane, 2017).

Mindfulness is defined as the practice of purposely bringing one’s attention in the present moment without judgment (Kabat-Zinn, 1990). One of mindfulness’ various conceptual delimitations was provided by Brown and Ryan, who define it as a psychological trait describing the tendency to be conscious in everyday life’s now moments (Brown & Ryan, 2003). Researchers have identified two main components that shape dispositional mindfulness: self-regulation of attention (i.e., focusing attention on the present moment) and orientation toward experience (i.e., having an open, conscious and acceptive mindset). According to previous studies, the second one would be the component that would have the most implications for good mental health (MacDonald & Baxter, 2017). At the same time, positive associations between mindfulness and people’s health have been consolidated. For example, several studies have shown positive associations between high levels of mindfulness as a trait and life satisfaction (Kong et al., 2014; Schutte & Malouff, 2011; Weinstein et al., 2009; Howell et al., 2008; Brown & Ryan, 2003). Furthermore, it has also been negatively related to aspects contrary to well-being or satisfaction with life, such as negative affect (Bajaj & Pande, 2016) as well as negative associations with anxiety or stress symptoms (Keng et al., 2011).

Concurrently, the focus has also been placed on the relationship between mindfulness and time perspective, providing evidence of the association between them (Drake et al., 2008). In general, it has been found that, in order to develop an optimal time perspective, one must be conscious of one’s own temporal perspective in adjusting flexibly to the demands of the environment (Stolarski et al., 2016). This is to say, it supposes the recruitment of attentional skills that regulates the focus of attention on temporal perspective. In other studies, this hypothesis was supported, and mindfulness was identified as a facilitator of this temporal optimal profile, which also resulted in higher levels of life satisfaction (Stolarski et al., 2016; Muro et al., 2017). Based on this theoretical background, the general aim of this study was to replicate previous studies and analyze the possible underlying mechanisms towards the relationship between temporal perspective and life satisfaction, testing the possible mediating role of mindfulness as a dispositional trait in a sample of students at the University of Girona. In this way, it will be possible to identify those factors that may be contributing to the well-being of university students to consider them in the planning of prevention and intervention strategies in this population.

Considering the main objective of the study and theoretical background presented, the specific objectives are: (1) analyze whether the fact of being more focused on the past, present, or future is associated with higher or lower levels of subjective well-being; (2) examine the potential connections between BTP, mindfulness, and levels of life satisfaction; and (3) explore the role of BTP in the relationship between mindfulness and life satisfaction.

## Method

### Sample

Three hundred forty-one healthy Catalan university students completed a series of self-report online questionnaires. The initial sample size was 356 but was reduced when 15 questionnaires with incomplete replies were excluded. Of these 341 participants, 263 self-described as women (77.1%) and 75 (22%) as men, whereas three (0.9%) identified as non-binary. Participants’ mean age was 23.62 years (SD = 5.02; age range: 18–52 years). In total, 88.6% (n = 302) of the sample were undergraduate students, and 11.4% (n = 39) were master’s students. Otherwise, a 47.8% of the participants were from the Faculty of Sciences (N = 163), 32.3% from the Faculty of Education and Psychology (N = 110), 12% from the Faculty of Law (N = 41), 5.9% from the Faculty of Arts and Letters (N = 20) and a 2.1% from other faculties of the university (N = 7). All members of the sample gave explicit consent to participate.

## Instruments

*Zimbardo Time Perspective Inventory* (ZTPI; Zimbardo & Boyd, 1999). To assess TP, the Spanish-adapted version of the Zimbardo Temporal Perspective Inventory (Díaz-Morales, 2006) was used. This questionnaire consists of 56 items and measures attitudes in relation to time on a Likert scale ranging from 1 to 5 (1 = *completely false*, 5 = *completely true*). Its structure is based on five factors: past negative; positive past; present hedonist; present fatalist; and future. It has demonstrated adequate validity and reliability, as well as easy administration (Zimbardo & Boyd, 1999). In this study, the Cronbach Alpha was 0.78 for Past Negative, 0.73 for Past Positive, 0.77 for Present Hedonistic and 0.54 for Present Fatalistic.

*Satisfaction With Life Scale* (SWLS; Diener et al., 1985). The Spanish version of the life satisfaction scale (Atienza et al., 2000) was used to measure life satisfaction. This instrument assesses life satisfaction globally, has been used in many other studies worldwide, and has been translated and validated in many languages (Muro et al., 2017). It comprises five items scored on a Likert scale ranging from 1 (*strongly disagree*) to 7 (*strongly agree*). The scale’s Spanish adaptation shows good fit and internal consistency (Atienza et al., 2000). In this study, the Cronbach Alpha was 0.85.

*Mindfulness Awareness Scale* (MAAS; Brown & Ryan, 2003). To assess mindfulness as a trait, the Spanish version of the Mindfulness Awareness Scale (Soler et al., 2012) was administrated. The MAAS is a simple, quick-administration scale that assesses an individual’s dispositional ability to be attentive and conscious toward the experience of the present moment in daily life. It consists of 15 items and is scored on a Likert scale ranging from 1 (*almost always*) to 6 (*almost never*). It also measures the frequency of the state of mindfulness without the need for previous training (Soler et al., 2012). In this study, the Cronbach Alpha was 0.80.

## Procedure

The Google Forms web application was used to administer all three questionnaires simultaneously. The data collection process had to be adapted to this format due to the COVID-19 pandemic. The questionnaires were disseminated via social networks. Several coordinators of studies at the University of Girona (in psychology, biotechnology, mechanical engineering, and social work) were also contacted with a request to disseminate the questionnaires to their students.

An informative text was prepared to inform respondents about the objectives of the study and the anonymous, confidential, and voluntary nature of participation, as well as confidentiality-related issues linked to the Catalan, Spanish and European Regulations on Data Protection and Guarantee of Digital Rights. This text provided the principal investigator’s email in case respondents wanted to contact the research team with any questions related to the study, asked whether they accepted the terms of participation, and prompted them to give their consent to participate. We ended the set of questions with a final open-ended question so that respondents could provide comments or suggestions. The answers obtained through the Google Forms survey were transferred to Microsoft Excel to facilitate their subsequent introduction into the SPSS statistics software (version 26). The study satisfied all the standards of the University of Girona, existing laws, and the APA code of ethics, including obtaining informed consent from all participants and collecting entirely anonymous data. Finally, convenience sampling was used to collect the data between December 2020 and January 2021.

### Data analysis

Data was analyzed using the statistical package IBM SPSS Statistics for Windows (version 26.0). First, descriptive analyses were performed to identify the means and standard deviations of the sample and normality was verified using Shapiro-Wilk test. Secondly, Cronbach’s alphas were calculated to obtain a reliability measure for all the questionnaires. Thirdly, differences regarding gender were analyzed by applying Student’s *t*-tests (normally distributed) or Mann-Whitney U-test (non-normally distributed). To describe the effect size, Cohen’s *d* (1988) was calculated for those significant results. Pearson’s correlations were carried out to analyze the possible associations between the study variables.

On the other hand, DBTP coefficient was calculated following Stolarski et al. (2011). This coefficient indicates how close or far a person is from having a BTP and is calculated according to the following formula: DBTP =

1 $$\sqrt{(oPN-ePN{)}^{2}+(oPP-ePP{)}^{2}+(oPF-ePF{)}^{2}+(oPH-ePH{)}^{2}+(oF-eF{)}^{2}}$$

where *o* represents “observed,” or the value obtained for each TP frame (*PN* = past negative; *PP* = past positive; *PF* = present fatalistic; *PH* = present hedonistic; *F* = future), and *e* stands for the expected optimal value for each TP. We defined the optimal scores following Zimbardo and Boyd’s proposal (www.thetimeparadox.com/surveys) so we assigned high scores on past positive (4.60) and future (4.00), moderately high on present hedonistic (3.90), and low on past negative (1.95) and present fatalistic (1.50). The closer to zero a coefficient is, the closer that person is to having a BTP. Pearson correlations were subsequently performed to analyze the relationships among questionnaire scores.

Finally, the PROCESS Macro for SPSS version 4.1 (Hayes, 2018) was used to investigate the potential mediating effect of DBTP on mindfulness and life satisfaction. Mindfulness was introduced as the independent variable, satisfaction with life as the dependent, and the BTP coefficient as the mediator. Otherwise, to test the simple mediation model, Model 4 was selected, as well as a bootstrap of 10.000 and a 95% confidence interval. Coefficients were standardized.

## Results

Since there were insufficient non-binary participants to conduct the analyses, the results were only compared between males and females. Means, standard deviations and mean comparison’s results for temporal perspectives, BTP scores, mindfulness as a trait (using the MAAS), and life satisfaction (using the SWLS) are shown at Table 2.

**Table 2:** *Differences for all measures according to gender*

	Male *N* = 75		Female *N* = 263		*t/z*	*p*	*d*
	**Mean**	**SD**	**Mean**	**SD**			
Age	22.28	3.42	22.02	4.53			
Past Negative	3.27	0.58	3.27	0.68	0.041	0.967	
Past Positive	3.56	0.62	3.73	0.56	-2.29	< 0.05*	0.291
Present Hedonistic	3.38	.51	3.44	0.49	− 0.939	0.348	
Present Fatalistic	2.60	0.51	2.69	0.48	-1.42	0.158	
Future	3.44	0.61	3.64	0.51	-2.89	< 0.05*	0.477
Balanced Time Perspective	2.40	0.68	2.33	0.60	0.963	0.336	
Mindfulness Awareness Scale	54.87	12.44	56.54	11.05	-1.13	0.261	
Life Satisfaction	20.63	6.91	20.97	6.61	− 0.380	0.705	

*Note*. *N* = 341.

The results obtained indicate that there were no differences regarding to gender in terms of satisfaction with life and dispositional mindfulness. On the other hand, there were significant differences in Past Positive (*t*(336) = -2.29, *p* < .05) and Future (*t*(336)= -2.89, *p* < .05), where women score higher compared to men.

Pearson’s correlations for all measures are shown at Table 3. First, it is observed that age has been positively correlated with MAAS (*r* = .140, *p* < .01) and negatively correlated with the present hedonistic (*r* = − .208, *p* = .01) scores. This would imply that the older we get, the more likely we are to be attentive and aware of the present moment experience, yet less focused on the hedonistic aspect of it.

**Table 3:** *Pearson’s correlations for all measures*

	1	2	3	4	5	6	7	8	9
**1. Age**	-								
**2. PN**	− 0.087	-							
**3. PP**	− 0.033	− 0.235^**^	-						
**4. PH**	− 0.202^**^	− 0.051	0.178^**^	-					
**5. PF**	− 0.053	0.163^**^	0.109^*^	0.361^**^	-				
**6. F**	,078	0.005	0.109*	− 0.298^**^	− 0.174^**^	-			
**7. MAAS**	0.142^**^	− 0.430^**^	0.173^**^	− 0.088	− 0.153^**^	0.224^**^	-		
**8. SWLS**	0.009	− 0.579^**^	0.391^**^	0.220^**^	0.020	0.143^**^	0.252^**^	-	
**9. BTP**	− 0.037	0.761^**^	− 0.549^**^	− 0.060	0.427^**^	− 0.263^**^	− 0.439^**^	− 0.565^**^	-

*Note*. PN = Past Negative; PP = Past Positive; PH = Present Hedonistic; PF = Present Fatalistic; F = Future; MAAS = Mindfulness Awareness Scale; SWLS = Satisfaction with Life Scale; BTP = Balanced Time Perspective. *p < .05, **p < .01

On the other hand, Past Positive correlated negatively with past negative (*r* = − .237, *p* = .01). The results also indicated a negative correlation between Past Negative perspective with both mindfulness (*r* = − .431, *p* = .01) and satisfaction with life (*r* = − .579, *p* = .01). In stark contrast, positive correlations were observed between Past Positive perspective and both mindfulness (*r* = .170, *p* = .01) and satisfaction with life (*r* = .388, *p* = .01).

Present Hedonistic perspective was uncorrelated with MAAS but positively associated with SWLS (*r* = .218, *p* = .01). In contrast, Present Fatalistic perspective was uncorrelated with SWLS but negatively correlated with MAAS (*r* = − .153, *p* = .01). Finally, Future perspective was positively correlated with both MAAS (*r* = .224, *p* = .01) and SWLS (*r* = .143, *p* = .01).

Regarding BTP, the results indicated significant negative correlations with MAAS (*r* = − .438, *p* = .01), SWLS (*r* = − .563, *p* = .01), Past Positive (*r* = − .548, *p* = .01) and Future (*r* = − .261, *p* = .01), as well as significant positive correlations with Past Negative (*r* = .762, *p* = .01) and Present Fatalistic (*r* = .428, *p* = .01). It should be noted that BTP scores the deviation from a theoretically “good” Balanced Time Perspective. As a result, these findings make sense when we consider that a greater BTP indicates a reduced capacity to balance across temporal perspectives.

**Fig. 1:**
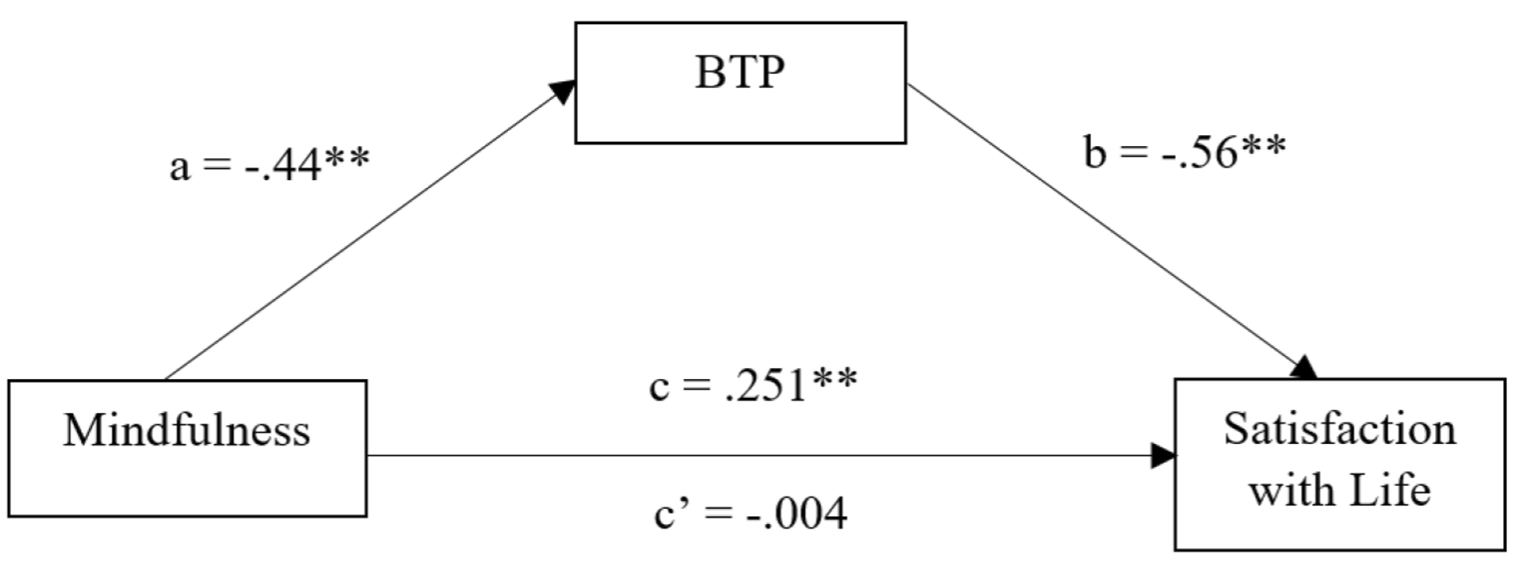
Illustration of the final mediation model *Note:* BTP = Balanced Time Perspective. a = direct effect of mindfulness on balanced time perspective; b = direct effect of balanced time perspective on satisfaction with life; c = total effect of mindfulness on satisfaction with life not accounting for balanced time perspective; c’= direct effect of mindfulness on satisfaction with life accounting for balanced time perspective. Model 4, bootstrap = 10.000, standardized coefficients.

Our last objective was to assess whether BTP could act as a mediator between Mindfulness as a trait and Satisfaction with Life. Results showed significant indirect effects of mindfulness as a trait on satisfaction with life through BTP. Specifically, results showed significant coefficients of paths *a*1 and *b1*, indicating a negative association between mindfulness and BTP (*β* = − 0.024, *p* < .001) and negative association between BTP and life satisfaction (*β* = -6.12, *p* < .001). Furthermore, the indirect effect for mindfulness on satisfaction with life through BTP was also significant (*β* = 0.247, bias corrected 95% confidence interval [BC95%CI] from 0.19 to 0.31), indicating significant mediation. The mediation model is visualized in Fig. 1.

Finally, the analyzes referring to the open question that appeared at the end of the questionnaire did not provide relevant information.

## Discussion

In the present study, several objectives were set for exploring the possible relationships among time perspective (TP), life satisfaction, and mindfulness as a trait. The first of our objectives was to analyze whether being more focused on the past, present, or future was associated with higher or lower levels of subjective well-being. The findings showed that people who tended to dwell on the bad past—that is, those who remember the past in a negative light, with feelings of failure, guilt, or pessimism—had lower levels of wellbeing. The opposite was true for people who focused on the positive aspects of their past. Thus, people who had focused on remembering the past in a warm and sentimental way tended to present higher levels of life satisfaction. These findings are consistent with those of other researchers for both the past negative (Muro et al., 2017; Zhang et al., 2013; Boniwell et al., 2010), and for the past positive (Pallini et al., 2018; Muro et al., 2017) relationships with well-being.

With reference to present TP, the results indicated that people who tended to focus on the hedonistic present reported higher levels of satisfaction with life, although the relevant correlation was low. The results of studies conducted in other countries also suggest this association (Seema & Sircova, 2013; Vowinckel, 2012). On the other hand, Present Fatalistic didn’t reveal any correlation with levels of subjective well-being. This result differs from those of studies carried out in other countries, although it does coincide with other results obtained in Mediterranean samples (Muro et al., 2017; Ortuño et al., 2015). This difference could be explained by variations regarding cultural and religious traditions so it may be interesting for future research to consider how these socio-historical factors might explain these differences.

On the other hand, in this study the alpha value obtained in Present Fatalistic subscale was very low. We should consider that similar results were obtained in Spanish validation of ZTPI (Díaz-Morales, 2006) and other studies with Spanish population (Muro et al., 2015). Therefore, the non-significant result could be explained by the low reliability obtained in this scale in this cultural context.

In relation to Future, there is some scientific consensus regarding its relationship with higher levels of global life satisfaction (Zhang et al., 2013; Boniwell et al., 2010). In the present study, this time perspective was positively correlated with reported levels of subjective well-being, but was the lowest of any correlations that was found. Psychologists have noted the benefits of future-oriented thinking, arguing that it is a motivator for engaging in healthy behaviors and improving well-being (see, e.g., Boyd & Zimbardo, 2005). These findings are therefore linked to a vision of the future based on goal-achievement, that’s to say, the future is understood from the executive planning capabilities. However, it should be noted that the dimension of the future also includes other aspects that are not considered in this definition (e.g., its negative view, as discussed below). Considering this, it would be interesting to consider this negative view in future studies.

Another goal in this research was to explore the relationships among BTP, mindfulness as a trait, and levels of life satisfaction. Zimbardo and Boyd (2008) conceived of BTP as an idealized mental framework that allows for flexible switching between temporal frameworks depending on task characteristics, demands, personal resources, and social assessments. The study findings showed that BTP was the most strongly correlated measure with satisfaction with life, reflecting the same pattern as in other studies (Boniwell et al., 2010; Drake et al., 2008; Muro et al., 2017; Stolarski et al., 2016; Zhang et al., 2013). In this sense, it is worth highlighting that BTP is regarded as the primary goal of Zimbardo, Sword, and Sword’s time perspective therapy to ensure well-being (Zimbardo et al., 2012).

Regarding the results obtained for the relationship between BTP and MAAS score, we observed that these findings were significantly negatively correlated. This would explain why people with profiles that are closer to optimal from a BTP could have an increased ability to pay attention to the present moment and regulate their attention. These results also coincide with those obtained in previous studies, reinforcing the hypothesis that mindfulness could be an intrinsic component of temporal balance (Muro et al., 2017; Stolarski et al., 2015). Thus, in some ways, the concept of mindfulness could presuppose full attention, and not only in the present but this ability would also imply an ability to attend to past events or possible future adverse events with a compassionate attitude (e.g., empathic and avoiding value judgments).

On the other hand, the results indicated a positive association between mindfulness and life satisfaction, which is consistent with past research (MacDonald & Baxter, 2017; Kong et al., 2014). Therefore, people who have a greater self-regulation capacity of attention in the present moment and are oriented towards experiences in an open and conscious way, would tend to feel more satisfied with their lives. It should be borne in mind that, in a high-performing and accelerated society in which we live, this ability could allow us to reconnect with ourselves, creating a sense of well-being (Crane, 2017).

Finally, the last objective was to investigate the role of BTP in the relationship between mindfulness and life satisfaction. Mechanisms by which mindfulness produces such healthy effects on mental health and well-being are not clear enough (MacDonald & Baxter, 2017). However, the results obtained would indicate that people who report higher levels of awareness and attention to the events of daily life would feel more satisfied with their lives. In addition, one of the underlying mechanisms that could explain this association would be the balanced temporal perspective (BTP). These results are consistent with previous studies, such as that of Stolarski et al. (2016), where it was identified that BTP acted as a mediator between mindfulness and life satisfaction in three samples using different measures for mindfulness. In addition, it has been pointed out that a balanced temporal perspective would be important for maintaining and amplifying well-being (Bohart, 1993) and would be in line with the 3P Model proposed by Durayappah (2011). This model proposes that subjective well-being has a temporal component, since not only is intended to pursue happiness (in the Future), but also to experience it (in the Present), as well as protect our previously obtained happiness (in the Past) (Durayappah, 2011). Likewise, the main goal of the Temporal Perspective Therapy (TPT) proposed by Sword, Sword and Zimbardo et al. (2012), is to work and make the person aware of their own temporal perspective, in order to develop an optimal or balanced temporal perspective. So, the results would indicate that the practice of mindfulness would become an individual disposition that would allow the development of the capacity for temporary self-regulation, fact that could be considered in TPT. In addition, mindfulness oriented towards the regulation of attention towards TP could be a support in psychological interventions, alleviate temporary negative biases, and give rise to a more optimal and healthy perspective (Muro et al., 2017).

Finally, Vowinckel et al. (2017) proposed a scale that contemplated the present eudaimonic, conceptualized through constructs from positive psychology such as mindfulness and flow. This conceptualization of the present would lead to self-realization through the development of self-skills and talents (Kabat-Zinn, 1990). In this sense, one might think that the present eudaimonic might be less pleasing than a hedonistic perspective, but instead it would produce more meaning and reflection (Durayappah, 2011). Through elaborating and validating this scale, the authors observed that this dimension showed the most associations with positive mental health in comparison with the other temporal perspectives contemplated in the ZTPI. These results suggest that mindfulness and flow are important components of achieving BTP. Therefore, it should be kept in mind that the conceptualization of the present eudaimonic has been a shortcoming in this study, and it would be interesting to incorporate it in future research.

## Implications of the study

The present study explored the relationships among mindfulness as a trait, satisfaction with life and time perspective. Results showed that balanced time perspective could act as a mediator between mindfulness and life satisfaction, confirming previous research in this area. These findings could contribute to reinforce the importance of time perspective in relation to subjective well-being, as well as being able to work with attention and conscience towards one’s own time perspective as an important element in university students.

On the other hand, this study could add support to the notion that the most associated time perspectives to psychological well-being are Past Negative (in a negative way) and balanced time perspective (in a positive way). In addition, it could also contribute to the consideration of time perspectives in mindfulness-based therapies in order to be aware about them and regulate those perspectives that could be negatively impacting student’s well-being.

## Limitations and Future Research

Despite its positive results, this study also has several limitations. First, it should be noted that this research used a cross-sectional design. On the other hand, the study design and statistical analysis did not allow us to control the variables nor to establish causal relationships between them. In addition, variable measurement tools can contribute to bias associated with self-report measures (e.g., social desirability, self-deception, and lack of understanding of questions asked). Furthermore, our sample size was limited, with a significant disproportion between men and women. It is also a homogeneous sample: most participants were university students from the University of Girona, and the difference between academic disciplines was not considered (given the substantial variety in the sample).

On the other hand, the benefits of the future temporal perspective are often highlighted, but the negative effects of focusing on the future have tended to be ignored in TP research. For example, feelings of loss of control, fear, and threats to the future have been shown to be important predictors of symptoms of post-traumatic stress disorder and depression in Yugoslav war survivors (Başoǧlu et al., 2002; Carelli et al., 2011) proposed that the dimension of the future should be expanded and better specified. They proposed an expanded version of the ZTPI that suggested a six-factor structure including the negative future dimension. Their results showed that the negative future is a central dimension of temporal perspective. Therefore, future research in this area might include the dimension of the future negative in the Catalan population because doing so could yield important results in relation to the field of TP and mental health. It would also be interesting to carry out more empirical studies in Mediterranean cultures because there are some contradictory results between samples and examine in more detail these cultural differences.

As previously stated, although there is an increasing number of reports on the relationship between TP and psychiatric problems, this research is still in its early stages. It would be useful to assess the many applications of these contributions regarding TP in therapeutic practice, in line with the proposals of Zimbardo, Sword, and Sword (Zimbardo et al., 2012). Finally, to address the limitation related to the temporality of the cross-sectional study, it would be interesting to carry out longitudinal studies where the variables are incorporated.

Finally, there are studies that have related the practice of mindfulness to some specific characteristics of time perception (Kramer et al., 2013). Future research on the potential association between temporal perspective and mindfulness practice as well as the potential processes underlying these associations may be taking into account.

## Conclusion

The literature highlights the importance of individuals’ attitudes in relation to the past, present, and future in various areas (Boniwell et al., 2010; Mello & Worrell, 2006; Henson et al., 2006). In addition, the psychological construction of time is an important basis for our understanding of our experiences in the world, including the form of our thoughts and our lives (Stolarski et al., 2011).

The present study using data from young Catalan university students, would support the idea that people who demonstrate an improved ability to pay attention to the present moment and regulate their attention, would be more satisfied with their lives. In addition, one of the mechanisms that could explain this relationship would be a more balanced time perspective. Thus, future research might propose interventions intended to balance the temporal perspective on this stage through the regulation of one’s attention in the present moment given that it would increase levels of well-being.

## Electronic supplementary material

Below is the link to the electronic supplementary material.

Supplementary Material 1
